# Supplementary material for: Predicting N6-Methyladenosine Sites in Multiple Tissues of Mammals through Ensemble Deep Learning
Source: Int J Mol Sci. 2022 Dec 7;23(24):15490. doi: 10.3390/ijms232415490 (PMC9778682; doi:10.3390/ijms232415490)
Supplement: Supplementary file 1 [file ijms-23-15490-s001.zip › ijms-2043144-supplementary.pdf]

International Journal of *Molecular Sciences*

MDPI

Supplementary Materials

Predicting N6-Methyladenosine Sites in Multiple Tissues of Mammals through Ensemble Deep Learning

Zhengtao Luo †, Liliang Lou †, Wangren Qiu, Zhaochun Xu * and Xuan Xiao *

[1] Computer Department, Jingdezhen Ceramic University, Jingdezhen 333403, China
* Correspondence: xuzhaochun@jcu.edu.cn (Z.X.); Xiaoxuan@jcu.edu.cn (X.X.)
† These authors contributed equally

Table S1. Performance comparison between different base-classifier combinations on liver training data of human using five-fold cross validation

Ensemble framework	Base-classifier combination	ACC (%)	Sn (%)	)Sp (%)	MCC	AUROC
Single classifier	(1) SVM	78.66	79.61	77.71	0.57	0.8681
	(2) KNN	77.51	70.16	84.85	0.56	0.8655
	(3) RF	78.82	80.87	76.77	0.58	0.8660
	(4) GB	78.83	80.03	77.64	0.58	0.8678
	(5) CNN	80.68	83.60	77.75	0.61	0.8885
	(6) FCN	78.72	78.21	79.23	0.57	0.8592
	(7) LSTM	80.35	83.37	77.33	0.61	0.8811
Ensemble across multiple models	(1)+(2)+(3)+(4)+(5)	79.84	80.87	78.82	0.60	0.8833
	(1)+(2)+(3)+(4)+(6)	79.57	79.08	80.07	0.59	0.8761
	(1)+(2)+(3)+(4)+(7)	79.67	80.30	79.04	0.59	0.8837
	(1)+(2)+(3)+(5)+(6)	79.65	79.12	80.18	0.59	0.8827
	(1)+(2)+(4)+(5)+(6)	79.92	79.31	80.52	0.59	0.8815
	(1)+(3)+(4)+(5)+(6)	80.07	81.59	78.55	0.60	0.8806
	(2)+(3)+(4)+(5)+(6)	80.14	79.38	80.90	0.60	0.8835
	(1)+(2)+(3)+(6)+(7)	79.63	79.31	79.95	0.59	0.8834
	(1)+(2)+(4)+(6)+(7)	79.80	79.54	80.07	0.60	0.8795
	(1)+(3)+(4)+(6)+(7)	79.86	79.38	80.33	0.60	0.8820
	(1)+(2)+(3)+(5)+(7)	80.28	81.55	79.01	0.61	0.8888
	(1)+(2)+(4)+(5)+(7)	80.37	80.30	80.45	0.61	0.8891
	(1)+(3)+(4)+(5)+(7)	80.24	82.84	77.64	0.61	0.8875
	(2)+(3)+(4)+(5)+(7)	80.33	80.68	79.99	0.61	0.8886
	(1)+(2)+(5)+(6)+(7)	80.52	81.13	79.92	0.61	0.8873
	(2)+(3)+(4)+(6)+(7)	80.30	79.95	80.64	0.61	0.8820
	(1)+(3)+(5)+(6)+(7)	80.14	81.17	79.12	0.60	0.8865
	(1)+(4)+(5)+(6)+(7)	80.33	82.16	78.51	0.61	0.8866
	(2)+(3)+(5)+(6)+(7)	80.13	79.69	80.56	0.60	0.8891
	(2)+(4)+(5)+(6)+(7)	80.14	79.57	80.71	0.60	0.8849
	(3)+(4)+(5)+(6)+(7)	80.50	81.70	79.31	0.60	0.8863
Ensemble with same kind of model	(1)+(1)+(1)+(1)+(1)	79.48	80.45	78.51	0.59	0.8696

(2)+(2)+(2)+(2)+(2)	78.04	71.26	84.81	0.57	0.8657
(3)+(3)+(3)+(3)+(3)	78.68	80.75	76.61	0.57	0.8673
(4)+(4)+(4)+(4)+(4)	79.18	80.60	77.75	0.58	0.8707
**(5)+(5)+(5)+(5)+(5)**	**81.55**	**84.13**	**78.97**	**0.63**	**0.8915**
(6)+(6)+(6)+(6)+(6)	78.36	73.61	83.11	0.57	0.8633
(7)+(7)+(7)+(7)+(7)	80.64	83.86	77.41	0.61	0.8885

Table S2. Performance comparison between different base-classifier combinations on kidney training data of human using five-fold cross validation

Ensemble framework	Base-classifier combination	ACC (%)	Sn (%)	Sp (%)	MCC	AUROC
Single classifier	(1) SVM	77.96	79.34	76.59	0.56	0.8586
	(2) KNN	76.95	73.20	80.70	0.54	0.8527
	(3) RF	77.55	81.33	73.76	0.55	0.8564
	(4) GB	78.29	80.08	76.50	0.56	0.8612
	(5) CNN	80.09	83.73	76.45	0.60	0.8821
	(6) FCN	76.72	72.72	80.72	0.53	0.8462
	(7) LSTM	79.08	81.26	76.89	0.58	0.8720
Ensemble across multiple models	(1)+(2)+(3)+(4)+(5)	79.11	81.00	77.22	0.58	0.8766
	(1)+(2)+(3)+(4)+(6)	78.30	79.14	77.46	0.56	0.8640
	(1)+(2)+(3)+(4)+(7)	79.25	81.15	77.35	0.58	0.8770
	(1)+(2)+(3)+(5)+(6)	79.09	79.86	78.31	0.58	0.8730
	(1)+(2)+(4)+(5)+(6)	78.97	79.36	78.57	0.57	0.8733
	(1)+(3)+(4)+(5)+(6)	79.14	80.54	77.74	0.58	0.8769
	(2)+(3)+(4)+(5)+(6)	79.22	80.52	77.92	0.58	0.8748
	(1)+(2)+(3)+(6)+(7)	79.18	80.21	78.14	0.58	0.8769
	(1)+(2)+(4)+(6)+(7)	79.06	79.56	78.55	0.58	0.8757
	(1)+(3)+(4)+(6)+(7)	78.66	78.57	78.75	0.57	0.8763
	(1)+(2)+(3)+(5)+(7)	79.66	81.09	78.22	0.59	0.8830
	(1)+(2)+(4)+(5)+(7)	79.80	81.02	78.57	0.59	0.8841
	(1)+(3)+(4)+(5)+(7)	79.63	82.47	76.80	0.59	0.8824
	(2)+(3)+(4)+(5)+(7)	78.84	82.14	77.55	0.58	0.8833
	(1)+(2)+(5)+(6)+(7)	79.31	79.30	79.32	0.58	0.8824
	(2)+(3)+(4)+(6)+(7)	79.29	80.65	77.92	0.59	0.8766
	(1)+(3)+(5)+(6)+(7)	79.65	81.05	78.25	0.59	0.8828
	(1)+(4)+(5)+(6)+(7)	79.71	81.29	78.14	0.60	0.8808
	(2)+(3)+(5)+(6)+(7)	80.05	81.48	78.62	0.59	0.8790
	(2)+(4)+(5)+(6)+(7)	80.00	80.35	79.65	0.59	0.8829
	(3)+(4)+(5)+(6)+(7)	79.63	81.70	77.57	0.56	0.8810
Ensemble with same kind of model	(1)+(1)+(1)+(1)+(1)	78.24	79.80	76.67	0.54	0.8602
	(2)+(2)+(2)+(2)+(2)	77.09	73.46	80.72	0.56	0.8547
	(3)+(3)+(3)+(3)+(3)	77.93	81.92	73.94	0.56	0.8577
	(4)+(4)+(4)+(4)+(4)	78.38	79.89	76.87	0.56	0.8623
	**(5)+(5)+(5)+(5)+(5)**	**80.67**	**84.89**	**76.45**	**0.62**	**0.8896**
	(6)+(6)+(6)+(6)+(6)	77.47	72.91	82.03	0.55	0.8555
	(7)+(7)+(7)+(7)+(7)	80.32	84.48	76.17	0.61	0.8837

Table S3. Performance comparison between different base-classifier combinations on brain training data of mouse using five-fold cross validation

Ensemble framework	Base-classifier combination	ACC (%)	Sn (%)	Sp (%)	MCC	AUROC
Single classifier	(1) SVM	77.22	79.26	75.18	0.54	0.8487
	(2) KNN	76.28	83.09	69.47	0.53	0.8442
	(3) RF	76.19	80.26	72.12	0.53	0.8406
	(4) GB	76.86	79.76	73.96	0.54	0.8492
	(5) CNN	79.56	81.86	77.27	0.59	0.8815
	(6) FCN	76.12	73.78	78.47	0.52	0.8057
	(7) LSTM	78.72	81.28	76.16	0.58	0.8708
Ensemble across multiple models	(1)+(2)+(3)+(4)+(5)	78.14	82.11	74.18	0.56	0.8703
	(1)+(2)+(3)+(4)+(6)	77.52	80.71	74.33	0.55	0.8559
	(1)+(2)+(3)+(4)+(7)	78.09	81.74	74.43	0.56	0.8704
	(1)+(2)+(3)+(5)+(6)	78.20	81.50	74.90	0.57	0.8636
	(1)+(2)+(4)+(5)+(6)	78.05	80.59	75.51	0.56	0.8669
	(1)+(3)+(4)+(5)+(6)	78.22	80.79	75.66	0.57	0.8705
	(2)+(3)+(4)+(5)+(6)	77.81	81.46	74.17	0.56	0.8699
	(1)+(2)+(3)+(6)+(7)	77.96	80.59	75.34	0.56	0.8653
	(1)+(2)+(4)+(6)+(7)	77.76	80.64	74.89	0.56	0.8692
	(1)+(3)+(4)+(6)+(7)	77.73	80.64	74.83	0.56	0.8673
	(1)+(2)+(3)+(5)+(7)	78.70	82.64	74.75	0.56	0.8781
	(1)+(2)+(4)+(5)+(7)	78.73	82.90	74.55	0.58	0.8787
	(1)+(3)+(4)+(5)+(7)	78.58	82.50	74.65	0.58	0.8770
	(2)+(3)+(4)+(5)+(7)	78.54	82.84	74.23	0.57	0.8780
	(1)+(2)+(5)+(6)+(7)	78.51	81.30	75.73	0.57	0.8775
	(2)+(3)+(4)+(6)+(7)	78.22	80.85	75.59	0.57	0.8692
	(1)+(3)+(5)+(6)+(7)	78.87	81.32	76.42	0.57	0.8785
	(1)+(4)+(5)+(6)+(7)	78.74	81.40	76.09	0.58	0.8786
	(2)+(3)+(5)+(6)+(7)	78.88	82.53	75.23	0.58	0.8755
	(2)+(4)+(5)+(6)+(7)	78.70	82.88	74.53	0.58	0.8778
	(3)+(4)+(5)+(6)+(7)	78.57	81.06	76.09	0.57	0.8774
Ensemble with same kind of model	(1)+(1)+(1)+(1)+(1)	76.88	78.70	75.05	0.54	0.8480
	(2)+(2)+(2)+(2)+(2)	76.36	83.44	69.27	0.53	0.8457
	(3)+(3)+(3)+(3)+(3)	76.44	80.64	72.25	0.53	0.8445
	(4)+(4)+(4)+(4)+(4)	76.87	79.61	74.13	0.54	0.8502
	**(5)+(5)+(5)+(5)+(5)**	**79.85**	**83.54**	**76.16**	**0.60**	**0.8847**
	(6)+(6)+(6)+(6)+(6)	76.77	72.77	80.77	0.54	0.8502
	(7)+(7)+(7)+(7)+(7)	79.55	81.18	77.92	0.59	0.8798

Table S4. Performance comparison between different base-classifier combinations on liver training data of mouse using five-fold cross validation

Ensemble framework	Base-classifier combination	ACC (%)	Sn (%)	Sp (%)	MCC	AUROC
Single classifier	(1) SVM	68.75	73.34	64.17	0.38	0.7507
	(2) KNN	67.31	79.51	55.12	0.36	0.7436
	(3) RF	68.24	75.95	60.54	0.37	0.7525
	(4) GB	68.09	74.52	61.65	0.36	0.7508
	(5) CNN	71.74	76.63	66.85	0.44	0.7911
	(6) FCN	67.32	65.96	68.69	0.35	0.7275
	(7) LSTM	71.04	80.33	61.75	0.43	0.79
Ensemble across multiple models	(1)+(2)+(3)+(4)+(5)	70.11	79.14	61.07	0.41	0.7902
	(1)+(2)+(3)+(4)+(6)	69.44	75.9	62.98	0.39	0.7624
	(1)+(2)+(3)+(4)+(7)	69.78	77.47	62.09	0.4	0.786
	(1)+(2)+(3)+(5)+(6)	70.17	76.58	63.76	0.41	0.7852
	(1)+(2)+(4)+(5)+(6)	69.71	76.87	62.55	0.4	0.7845
	(1)+(3)+(4)+(5)+(6)	69.95	76.09	63.8	0.4	0.7853
	(2)+(3)+(4)+(5)+(6)	70.23	77.23	63.22	0.41	0.7833
	(1)+(2)+(3)+(6)+(7)	69.56	77.16	61.96	0.4	0.7845
	(1)+(2)+(4)+(6)+(7)	70.17	77.86	62.47	0.41	0.7834
	(1)+(3)+(4)+(6)+(7)	69.74	76.63	62.86	0.4	0.7829
	(1)+(2)+(3)+(5)+(7)	70.97	79.31	62.62	0.43	0.8018
	(1)+(2)+(4)+(5)+(7)	71.13	79.29	62.98	0.43	0.804
	(1)+(3)+(4)+(5)+(7)	70.92	79.19	62.64	0.42	0.8002
	(2)+(3)+(4)+(5)+(7)	71.15	80.26	62.04	0.43	0.8052
	(1)+(2)+(5)+(6)+(7)	70.57	77.96	63.17	0.42	0.7984
	(2)+(3)+(4)+(6)+(7)	70.11	77.5	62.71	0.41	0.788
	(1)+(3)+(5)+(6)+(7)	70.68	76.94	64.41	0.42	0.8
	(1)+(4)+(5)+(6)+(7)	71.21	75.73	66.68	0.43	0.8017
	(2)+(3)+(5)+(6)+(7)	71.56	80.35	62.76	0.44	0.8054
	(2)+(4)+(5)+(6)+(7)	71.09	78.73	63.44	0.43	0.7994
	(3)+(4)+(5)+(6)+(7)	70.71	77.18	64.24	0.42	0.8014
Ensemble with same kind of model	(1)+(1)+(1)+(1)+(1)	68.53	72.83	64.24	0.37	0.7518
	(2)+(2)+(2)+(2)+(2)	67.58	80.55	54.61	0.36	0.7461
	(3)+(3)+(3)+(3)+(3)	68.45	76.12	60.78	0.37	0.756
	(4)+(4)+(4)+(4)+(4)	68.64	74.84	62.45	0.38	0.7562
	**(5)+(5)+(5)+(5)+(5)**	**73.53**	**84.01**	**63.05**	**0.48**	**0.8181**
	(6)+(6)+(6)+(6)+(6)	67.53	67.92	67.14	0.35	0.7493
	(7)+(7)+(7)+(7)+(7)	72.38	80.98	63.78	0.45	0.8016

Table S5. Performance comparison between different base-classifier combinations on kidney training data of mouse using five-fold cross validation

Ensemble framework	Base-classifier combination	ACC (%)	Sn (%)	Sp (%)	MCC	AUROC
Single classifier	(1) SVM	79.21	80.93	77.49	0.58	0.8707
	(2) KNN	79.21	82.17	76.25	0.59	0.8687
	(3) RF	78.4	81.74	75.06	0.57	0.8608
	(4) GB	79.03	81.31	76.75	0.58	0.8677
	(5) CNN	81.09	83.76	78.42	0.62	0.8897
	(6) FCN	77.42	74.17	80.67	0.55	0.8438
	(7) LSTM	80.86	83.03	78.7	0.62	0.8912
Ensemble across multiple models	(1)+(2)+(3)+(4)+(5)	80.41	82.85	77.97	0.61	0.8862
	(1)+(2)+(3)+(4)+(6)	79.79	81.63	77.94	0.6	0.8751
	(1)+(2)+(3)+(4)+(7)	80.12	82.29	77.94	0.6	0.8854
	(1)+(2)+(3)+(5)+(6)	79.98	82.11	77.84	0.6	0.8855
	(1)+(2)+(4)+(5)+(6)	79.98	81.41	78.55	0.6	0.8858
	(1)+(3)+(4)+(5)+(6)	80.22	81.46	78.98	0.6	0.8834
	(2)+(3)+(4)+(5)+(6)	79.89	81.1	78.67	0.6	0.8843
	(1)+(2)+(3)+(6)+(7)	80.2	82.04	78.37	0.6	0.8851
	(1)+(2)+(4)+(6)+(7)	80.36	81.36	79.36	0.61	0.8856
	(1)+(3)+(4)+(6)+(7)	80.08	81.68	78.47	0.6	0.885
	(1)+(2)+(3)+(5)+(7)	80.51	83.53	77.49	0.61	0.8935
	(1)+(2)+(4)+(5)+(7)	80.91	83.28	78.55	0.62	0.8933
	(1)+(3)+(4)+(5)+(7)	80.14	82.14	78.14	0.6	0.8894
	(2)+(3)+(4)+(5)+(7)	80.99	83.76	78.22	0.62	0.8947
	(1)+(2)+(5)+(6)+(7)	80.56	82.75	78.37	0.61	0.8926
	(2)+(3)+(4)+(6)+(7)	80.24	82.6	77.89	0.61	0.8863
	(1)+(3)+(5)+(6)+(7)	80.86	82.8	78.93	0.62	0.8909
	(1)+(4)+(5)+(6)+(7)	80.89	82.54	79.23	0.62	0.8891
	(2)+(3)+(5)+(6)+(7)	80.79	82.52	79.05	0.62	0.8933
	(2)+(4)+(5)+(6)+(7)	80.76	82.19	79.33	0.62	0.8929
	(3)+(4)+(5)+(6)+(7)	81.1	82.72	79.48	0.62	0.8924
Ensemble with same kind of model	(1)+(1)+(1)+(1)+(1)	79.48	81.25	77.71	0.59	0.871
	(2)+(2)+(2)+(2)+(2)	78.93	81.86	75.99	0.58	0.8685
	(3)+(3)+(3)+(3)+(3)	78.75	82.27	75.23	0.58	0.8631
	(4)+(4)+(4)+(4)+(4)	79.38	81.2	77.56	0.59	0.8688
	**(5)+(5)+(5)+(5)+(5)**	**81.96**	**83.51**	**80.42**	**0.64**	**0.9008**
	(6)+(6)+(6)+(6)+(6)	78.17	73.06	83.28	0.57	0.8596
	(7)+(7)+(7)+(7)+(7)	81.23	83	79.46	0.62	0.8952

Table S6. Performance comparison between different base-classifier combinations on heart training data of mouse using five-fold cross validation

Ensemble framework	Base-classifier combination	ACC (%)	Sn (%)	Sp (%)	MCC	AUROC
Single classifier	(1) SVM	71.29	75.6	66.97	0.43	0.7823
	(2) KNN	69.92	81.64	58.2	0.41	0.7765
	(3) RF	71.42	77.19	65.65	0.43	0.7828
	(4) GB	70.74	74.78	66.7	0.42	0.7784
	(5) CNN	74.24	82.51	65.97	0.49	0.8228
	(6) FCN	68.97	66.33	71.6	0.38	0.7559
	(7) LSTM	74.67	80.28	69.06	0.5	0.82
Ensemble across multiple models	(1)+(2)+(3)+(4)+(5)	72.24	79.51	64.97	0.45	0.8103
	(1)+(2)+(3)+(4)+(6)	72.44	77.42	67.47	0.45	0.7869
	(1)+(2)+(3)+(4)+(7)	71.83	78.37	65.29	0.44	0.8084
	(1)+(2)+(3)+(5)+(6)	72.17	78.37	65.97	0.45	0.8117
	(1)+(2)+(4)+(5)+(6)	71.51	76.78	66.24	0.43	0.8038
	(1)+(3)+(4)+(5)+(6)	72.31	77.01	67.61	0.45	0.807
	(2)+(3)+(4)+(5)+(6)	72.42	78.83	66.02	0.45	0.8111
	(1)+(2)+(3)+(6)+(7)	72.51	76.37	68.65	0.45	0.8084
	(1)+(2)+(4)+(6)+(7)	71.58	77.42	65.74	0.43	0.8036
	(1)+(3)+(4)+(6)+(7)	72.06	76.6	67.51	0.44	0.8069
	(1)+(2)+(3)+(5)+(7)	73.1	80.55	65.65	0.47	0.8243
	(1)+(2)+(4)+(5)+(7)	72.65	79.51	65.79	0.46	0.8161
	(1)+(3)+(4)+(5)+(7)	73.13	79.96	66.29	0.47	0.8195
	(2)+(3)+(4)+(5)+(7)	72.72	80.28	65.15	0.46	0.8245
	(1)+(2)+(5)+(6)+(7)	72.85	79.33	66.38	0.46	0.8181
	(2)+(3)+(4)+(6)+(7)	72.44	78.78	66.11	0.45	0.8077
	(1)+(3)+(5)+(6)+(7)	73.42	75.24	71.6	0.47	0.8196
	(1)+(4)+(5)+(6)+(7)	72.94	75.33	70.56	0.46	0.8141
	(2)+(3)+(5)+(6)+(7)	73.17	81.42	64.93	0.47	0.8166
	(2)+(4)+(5)+(6)+(7)	73.42	79.92	66.92	0.47	0.8233
	(3)+(4)+(5)+(6)+(7)	73.01	79.01	67.01	0.46	0.8169
Ensemble with same kind of model	(1)+(1)+(1)+(1)+(1)	70.81	74.28	67.33	0.42	0.7784
	(2)+(2)+(2)+(2)+(2)	70.06	82.37	57.75	0.41	0.7801
	(3)+(3)+(3)+(3)+(3)	71.56	77.74	65.38	0.43	0.7828
	(4)+(4)+(4)+(4)+(4)	70.76	74.92	66.61	0.42	0.7781
	**(5)+(5)+(5)+(5)+(5)**	**75.31**	**81.69**	**68.92**	**0.51**	**0.8350**
	(6)+(6)+(6)+(6)+(6)	69.22	67.38	71.06	0.38	0.7754
	(7)+(7)+(7)+(7)+(7)	74.47	79.42	69.51	0.49	0.8263

Table S7. Performance comparison between different base-classifier combinations on testis training data of mouse using five-fold cross validation

Ensemble framework	Base-classifier combination	ACC (%)	Sn (%)	Sp (%)	MCC	AUROC
Single classifier	(1) SVM	72.97	76.52	69.41	0.46	0.8039
	(2) KNN	72.15	83.51	60.78	0.45	0.7973
	(3) RF	72.63	78.39	66.86	0.46	0.7931
	(4) GB	72.48	76.8	68.15	0.45	0.7988
	(5) CNN	76.45	83.28	69.62	0.53	0.8457
	(6) FCN	71.2	70.13	72.28	0.42	0.7889
	(7) LSTM	75.64	81.24	70.04	0.52	0.8348
Ensemble across multiple models	(1)+(2)+(3)+(4)+(5)	74.09	80.45	67.73	0.49	0.8301
	(1)+(2)+(3)+(4)+(6)	73.38	79.05	67.71	0.47	0.8064
	(1)+(2)+(3)+(4)+(7)	74.33	81.18	67.47	0.49	0.8316
	(1)+(2)+(3)+(5)+(6)	74.12	79.43	68.81	0.49	0.8282
	(1)+(2)+(4)+(5)+(6)	74.27	80.18	68.37	0.49	0.8291
	(1)+(3)+(4)+(5)+(6)	73.94	78.27	69.62	0.48	0.8261
	(2)+(3)+(4)+(5)+(6)	74.02	79.16	68.88	0.48	0.8287
	(1)+(2)+(3)+(6)+(7)	74.3	79.86	68.75	0.49	0.833
	(1)+(2)+(4)+(6)+(7)	73.82	79.03	68.6	0.48	0.8281
	(1)+(3)+(4)+(6)+(7)	74.4	78.88	69.92	0.49	0.8301
	(1)+(2)+(3)+(5)+(7)	75.13	82.32	67.94	0.51	0.8455
	(1)+(2)+(4)+(5)+(7)	74.69	81.54	67.84	0.5	0.8397
	(1)+(3)+(4)+(5)+(7)	75.04	80.03	70.04	0.5	0.844
	(2)+(3)+(4)+(5)+(7)	75.03	82.39	67.67	0.51	0.8444
	(1)+(2)+(5)+(6)+(7)	74.93	80.96	68.9	0.5	0.8444
	(2)+(3)+(4)+(6)+(7)	73.71	80.05	67.37	0.48	0.8308
	(1)+(3)+(5)+(6)+(7)	74.86	78.95	70.77	0.5	0.8395
	(1)+(4)+(5)+(6)+(7)	75.2	78.73	71.66	0.51	0.8414
	(2)+(3)+(5)+(6)+(7)	75.39	82.52	68.26	0.51	0.8417
	(2)+(4)+(5)+(6)+(7)	75.27	82.01	68.54	0.51	0.8442
	(3)+(4)+(5)+(6)+(7)	74.95	79.46	70.45	0.5	0.8391
Ensemble with same kind of model	(1)+(1)+(1)+(1)+(1)	73.34	77.06	69.62	0.47	0.8035
	(2)+(2)+(2)+(2)+(2)	71.79	83.2	60.38	0.45	0.8003
	(3)+(3)+(3)+(3)+(3)	72.49	78.18	66.79	0.45	0.7958
	(4)+(4)+(4)+(4)+(4)	72.12	76.86	67.37	0.44	0.7963
	**(5)+(5)+(5)+(5)+(5)**	**76.90**	**85.70**	**68.09**	**0.55**	**0.8522**
	(6)+(6)+(6)+(6)+(6)	72.35	72.34	72.36	0.45	0.8037
	(7)+(7)+(7)+(7)+(7)	76.69	81.54	71.85	0.54	0.8489

Table S8. Performance comparison between different base-classifier combinations on brain training data of rat using five-fold cross validation

Ensemble framework	Base-classifier combination	ACC (%)	Sn (%)	Sp (%)	MCC	AUROC
Single classifier	(1) SVM	73.98	76.87	71.09	0.48	0.8194
	(2) KNN	73.66	78.23	69.09	0.48	0.8184
	(3) RF	74.89	79.68	70.11	0.5	0.8189
	(4) GB	74.66	77.47	71.85	0.49	0.8261
	(5) CNN	76.7	75.09	78.32	0.53	0.8545
	(6) FCN	71.92	67.39	76.45	0.44	0.7871
	(7) LSTM	76.87	78.19	75.55	0.54	0.8494
Ensemble across multiple models	(1)+(2)+(3)+(4)+(5)	75.62	79.04	72.19	0.51	0.8436
	(1)+(2)+(3)+(4)+(6)	74.62	77.93	71.3	0.49	0.8296
	(1)+(2)+(3)+(4)+(7)	75.79	79.46	72.11	0.52	0.8442
	(1)+(2)+(3)+(5)+(6)	75.28	78.32	72.24	0.51	0.8425
	(1)+(2)+(4)+(5)+(6)	74.98	78.57	71.39	0.5	0.8413
	(1)+(3)+(4)+(5)+(6)	76.17	80.27	72.07	0.53	0.8413
	(2)+(3)+(4)+(5)+(6)	75.6	79.93	71.26	0.51	0.8413
	(1)+(2)+(3)+(6)+(7)	75.36	79.21	71.51	0.51	0.8447
	(1)+(2)+(4)+(6)+(7)	75.04	78.23	71.85	0.5	0.8401
	(1)+(3)+(4)+(6)+(7)	75.38	77.85	72.92	0.51	0.8433
	(1)+(2)+(3)+(5)+(7)	76.17	78.66	73.68	0.52	0.851
	(1)+(2)+(4)+(5)+(7)	75.34	79.12	71.56	0.51	0.8515
	(1)+(3)+(4)+(5)+(7)	75.94	79.68	72.19	0.52	0.8511
	(2)+(3)+(4)+(5)+(7)	76.06	79.85	72.28	0.52	0.8542
	(1)+(2)+(5)+(6)+(7)	75.57	78.74	72.41	0.51	0.8515
	(2)+(3)+(4)+(6)+(7)	75.34	78.95	71.73	0.51	0.8418
	(1)+(3)+(5)+(6)+(7)	76.87	79.76	73.98	0.54	0.8473
	(1)+(4)+(5)+(6)+(7)	76.34	78.23	74.45	0.53	0.8515
	(2)+(3)+(5)+(6)+(7)	76.62	79.97	73.26	0.53	0.8501
	(2)+(4)+(5)+(6)+(7)	76.45	79.59	73.3	0.53	0.8518
	(3)+(4)+(5)+(6)+(7)	76.7	80.57	72.83	0.54	0.8511
Ensemble with same kind of model	(1)+(1)+(1)+(1)+(1)	74.4	77.08	71.73	0.49	0.8229
	(2)+(2)+(2)+(2)+(2)	73.62	78.78	68.45	0.47	0.8195
	(3)+(3)+(3)+(3)+(3)	74.94	80.27	69.6	0.5	0.8241
	(4)+(4)+(4)+(4)+(4)	74.66	77.25	72.07	0.49	0.823
	**(5)+(5)+(5)+(5)+(5)**	**77.27**	**81.80**	**72.75**	**0.55**	**0.8580**
	(6)+(6)+(6)+(6)+(6)	72.87	68.07	77.68	0.46	0.8196
	(7)+(7)+(7)+(7)+(7)	77.26	80.48	74.11	0.55	0.8557

Table S9. Performance comparison between different base-classifier combinations on liver training data of rat using five-fold cross validation

Ensemble framework	Base-classifier combination	ACC (%)	Sn (%)	Sp (%)	MCC	AUROC
Single classifier	(1) SVM	79.46	82.41	76.5	0.59	0.8703
	(2) KNN	79.51	83.03	75.99	0.59	0.8709
	(3) RF	79.57	84.34	74.8	0.59	0.8616
	(4) GB	79.97	82.58	77.36	0.6	0.871
	(5) CNN	81.36	81.56	81.16	0.63	0.8887
	(6) FCN	77.78	76.22	79.34	0.56	0.8194
	(7) LSTM	80.28	83.03	77.53	0.61	0.8782
Ensemble across multiple models	(1)+(2)+(3)+(4)+(5)	80.85	83.71	77.98	0.62	0.8846
	(1)+(2)+(3)+(4)+(6)	80.25	82.29	78.21	0.61	0.8739
	(1)+(2)+(3)+(4)+(7)	80.76	84.34	77.19	0.62	0.8855
	(1)+(2)+(3)+(5)+(6)	80.36	83.48	77.24	0.61	0.8808
	(1)+(2)+(4)+(5)+(6)	80.31	82.12	78.49	0.61	0.8797
	(1)+(3)+(4)+(5)+(6)	80.9	84.28	77.53	0.62	0.8847
	(2)+(3)+(4)+(5)+(6)	81.02	83.09	78.94	0.62	0.8831
	(1)+(2)+(3)+(6)+(7)	80.62	84	77.24	0.61	0.8814
	(1)+(2)+(4)+(6)+(7)	80.87	83.09	78.66	0.62	0.8813
	(1)+(3)+(4)+(6)+(7)	80.99	83.48	78.49	0.62	0.8843
	(1)+(2)+(3)+(5)+(7)	81.24	85.53	76.96	0.63	0.8875
	(1)+(2)+(4)+(5)+(7)	81.21	83.88	78.55	0.63	0.8909
	(1)+(3)+(4)+(5)+(7)	81.1	84.05	78.15	0.62	0.8888
	(2)+(3)+(4)+(5)+(7)	80.87	83.77	77.98	0.62	0.888
	(1)+(2)+(5)+(6)+(7)	81.13	83.71	78.55	0.62	0.8854
	(2)+(3)+(4)+(6)+(7)	80.62	82.69	78.55	0.61	0.8807
	(1)+(3)+(5)+(6)+(7)	81.24	82.86	79.63	0.63	0.8845
	(1)+(4)+(5)+(6)+(7)	80.48	81.16	79.8	0.61	0.8845
	(2)+(3)+(5)+(6)+(7)	80.93	83.31	78.55	0.62	0.8857
	(2)+(4)+(5)+(6)+(7)	81.41	83.77	79.06	0.63	0.89
	(3)+(4)+(5)+(6)+(7)	81.24	84.96	77.53	0.63	0.8863
Ensemble with same kind of model	(1)+(1)+(1)+(1)+(1)	79.88	82.97	76.79	0.6	0.8744
	(2)+(2)+(2)+(2)+(2)	79.88	83.09	76.67	0.6	0.8753
	(3)+(3)+(3)+(3)+(3)	79.94	84.9	74.97	0.6	0.8623
	(4)+(4)+(4)+(4)+(4)	79.8	82.24	77.36	0.6	0.8696
	**(5)+(5)+(5)+(5)+(5)**	**81.81**	**82.24**	**81.38**	**0.64**	**0.8949**
	(6)+(6)+(6)+(6)+(6)	78.06	72.59	83.54	0.56	0.8633
	(7)+(7)+(7)+(7)+(7)	80.39	81.16	79.63	0.61	0.8827

Table S10. Performance comparison between different base-classifier combinations on kidney training data of rat using five-fold cross validation

Ensemble framework	Base-classifier combination	ACC (%)	Sn (%)	Sp (%)	MCC	AUROC
Single classifier	(1) SVM	81.07	82.52	79.61	0.62	0.8844
	(2) KNN	80.5	79.38	81.62	0.61	0.8798
	(3) RF	80.34	83.28	77.4	0.61	0.8747
	(4) GB	80.54	82.78	78.3	0.61	0.8832
	(5) CNN	82.45	83.11	81.79	0.65	0.9031
	(6) FCN	80.18	78.04	82.32	0.6	0.8633
	(7) LSTM	81.85	86.48	77.22	0.64	0.899
Ensemble across multiple models	(1)+(2)+(3)+(4)+(5)	81.66	83.51	79.81	0.63	0.8971
	(1)+(2)+(3)+(4)+(6)	81.37	81.71	81.04	0.63	0.8871
	(1)+(2)+(3)+(4)+(7)	81.62	83.25	79.99	0.63	0.8977
	(1)+(2)+(3)+(5)+(6)	81.58	81.21	81.94	0.63	0.8959
	(1)+(2)+(4)+(5)+(6)	81.28	81.53	81.04	0.63	0.895
	(1)+(3)+(4)+(5)+(6)	81.55	82.87	80.22	0.63	0.892
	(2)+(3)+(4)+(5)+(6)	81.78	82.41	81.15	0.64	0.8942
	(1)+(2)+(3)+(6)+(7)	81.87	82.93	80.8	0.64	0.8936
	(1)+(2)+(4)+(6)+(7)	81.26	82.44	80.08	0.63	0.8975
	(1)+(3)+(4)+(6)+(7)	81.68	83.16	80.19	0.63	0.8963
	(1)+(2)+(3)+(5)+(7)	82.26	84.79	79.73	0.65	0.9034
	(1)+(2)+(4)+(5)+(7)	81.91	83.75	80.08	0.64	0.9025
	(1)+(3)+(4)+(5)+(7)	82.48	84.36	80.6	0.65	0.9024
	(2)+(3)+(4)+(5)+(7)	82.42	85.03	79.81	0.65	0.9036
	(1)+(2)+(5)+(6)+(7)	82	82.58	81.42	0.64	0.9002
	(2)+(3)+(4)+(6)+(7)	81.97	83.02	80.92	0.64	0.8931
	(1)+(3)+(5)+(6)+(7)	82	83.37	80.63	0.64	0.8994
	(1)+(4)+(5)+(6)+(7)	82.2	83.63	80.77	0.64	0.899
	(2)+(3)+(5)+(6)+(7)	82.36	82.64	82.09	0.65	0.9032
	(2)+(4)+(5)+(6)+(7)	82.03	82.49	81.56	0.64	0.9018
	(3)+(4)+(5)+(6)+(7)	81.91	84.01	79.81	0.64	0.9013
Ensemble with same kind of model	(1)+(1)+(1)+(1)+(1)	80.69	82.09	79.29	0.61	0.8837
	(2)+(2)+(2)+(2)+(2)	80.7	80.16	81.24	0.61	0.8821
	(3)+(3)+(3)+(3)+(3)	80.16	83.34	76.99	0.6	0.8754
	(4)+(4)+(4)+(4)+(4)	80.19	82.32	78.07	0.6	0.8809
	**(5)+(5)+(5)+(5)+(5)**	**82.97**	**82.90**	**83.05**	**0.66**	**0.9061**
	(6)+(6)+(6)+(6)+(6)	80.1	74.42	85.79	0.61	0.8799
	(7)+(7)+(7)+(7)+(7)	82.7	86.54	78.85	0.66	0.905

Table S11: Five-fold cross-validation result comparison

Species	Methods	Tissues	ACC	Sn	Sp	MCC	AUC
Human	iRNA-m6A	Brain	71.26	74.79	66.19	0.41	0.7756
	m6A-TS-CNN	Brain	72.53	75.35	69.71	0.4523	0.8029
	TS-m6A-DL	Brain	75.07	81.91	68.23	0.5068	0.8262
	im6APred	Brain	74.23	82.48	65.99	0.49	0.8241
	iRNA-m6A	Kidney	78.99	80.85	76.34	0.57	0.8634
	m6A-TS-CNN	Kidney	79.98	81.70	78.25	0.6006	0.8781
	TS-m6A-DL	Kidney	80.99	83.93	78.04	0.6211	0.8904
	im6APred	Kidney	80.67	84.89	76.45	0.62	0.8896
	iRNA-m6A	Liver	80.13	81.32	78.13	0.59	0.8738
	m6A-TS-CNN	Liver	79.94	80.18	79.69	0.5992	0.8811
	TS-m6A-DL	Liver	83.35	85.95	80.75	0.6684	0.9135
	im6APred	Liver	81.55	84.13	78.98	0.63	0.8915
Rat	iRNA-m6A	Brain	75.96	77.00	73.47	0.50	0.8282
	m6A-TS-CNN	Brain	76.64	79.04	74.23	0.5379	0.8469
	TS-m6A-DL	Brain	79.55	82.27	76.82	0.5922	0.8758
	im6APred	Brain	77.27	81.80	72.75	0.55	0.8580
	iRNA-m6A	Kidney	81.78	82.46	80.05	0.63	0.8877
	m6A-TS-CNN	Kidney	82.46	84.15	80.77	0.6500	0.9017
	TS-m6A-DL	Kidney	83.41	84.00	82.81	0.6683	0.9066
	im6APred	Kidney	82.97	82.90	83.05	0.66	0.9061
	iRNA-m6A	Liver	80.90	83.09	76.33	0.60	0.8766
	m6A-TS-CNN	Liver	80.59	81.56	79.63	0.6126	0.8830
	TS-m6A-DL	Liver	83.48	83.47	83.48	0.6706	0.9025
	im6APred	Liver	81.81	82.24	81.38	0.64	0.8949
Mouse	iRNA-m6A	Brain	78.75	79.32	76.90	0.58	0.8701
	m6A-TS-CNN	Brain	78.67	81.50	75.85	0.5749	0.8705
	TS-m6A-DL	Brain	79.85	81.34	78.36	0.5974	0.8831
	im6APred	Brain	79.85	83.54	76.16	0.60	0.8847
	iRNA-m6A	Heart	72.76	75.24	68.97	0.44	0.7948
	m6A-TS-CNN	Heart	72.99	78.37	67.60	0.4633	0.8115
	TS-m6A-DL	Heart	78.23	81.50	74.96	0.5664	0.8504
	im6APred	Heart	75.31	81.69	68.92	0.51	0.8350
	iRNA-m6A	Kidney	79.98	82.60	77.31	0.60	0.8726
	m6A-TS-CNN	Kidney	80.46	79.91	81.00	0.6094	0.8842
	TS-m6A-DL	Kidney	82.22	82.59	81.86	0.6451	0.9079
	im6APred	Kidney	81.96	83.51	80.42	0.64	0.9008
	iRNA-m6A	Liver	70.59	74.93	65.59	0.41	0.7743
	m6A-TS-CNN	Liver	71.32	72.39	70.24	0.4288	0.7953
	TS-m6A-DL	Liver	75.06	79.53	70.60	0.5044	0.8288

	im6APred	Liver	73.53	84.01	63.05	0.48	0.8181
	iRNA-m6A	Testis	74.40	78.14	70.02	0.48	0.8156
	m6A-TS-CNN	Testis	75.41	75.21	75.61	0.5090	0.8380
	TS-m6A-DL	Testis	77.56	81.79	73.33	0.5544	0.8630
	im6APred	Testis	76.90	85.70	68.09	0.54	0.8522

Table S12: Independent result comparison

Species	Methods	Tissues	ACC	Sn	Sp	MCC	AUC
Human	iRNA-m6A	Brain	71.10	69.50	72.98	0.42	0.7845
	m6A-TS-CNN	Brain	72.69	75.17	70.20	0.4543	0.8056
	TS-m6A-DL	Brain	73.84	81.23	66.46	0.4822	0.8097
	im6APred	Brain	74.38	80.41	68.35	0.49	0.8290
	iRNA-m6A	Kidney	77.76	77.13	78.42	0.56	0.8565
	m6A-TS-CNN	Kidney	79.24	79.95	78.53	0.5848	0.8727
	TS-m6A-DL	Kidney	80.20	80.45	79.96	0.6042	0.8802
	im6APred	Kidney	80.59	87.29	73.89	0.61	0.8892
	iRNA-m6A	Liver	79.01	78.19	79.87	0.58	0.8681
	m6A-TS-CNN	Liver	79.92	84.81	75.02	0.6012	0.8805
	TS-m6A-DL	Liver	80.56	82.04	79.88	0.6115	0.8784
	im6APred	Liver	81.21	82.61	79.80	0.62	0.8909
Rat	iRNA-m6A	Brain	75.14	73.93	76.48	0.50	0.8265
	m6A-TS-CNN	Brain	76.95	78.05	75.84	0.5391	0.8516
	TS-m6A-DL	Brain	77.28	81.32	73.24	0.5475	0.8543
	im6APred	Brain	77.20	87.32	67.08	0.56	0.8680
	iRNA-m6A	Kidney	81.42	80.18	82.77	0.63	0.8968
	m6A-TS-CNN	Kidney	82.72	84.85	80.59	0.6550	0.9077
	TS-m6A-DL	Kidney	83.27	85.22	81.32	0.6660	0.9083
	im6APred	Kidney	83.92	84.91	82.93	0.68	0.9173
	iRNA-m6A	Liver	79.85	77.74	82.31	0.60	0.8761
	m6A-TS-CNN	Liver	80.22	84.51	75.94	0.6067	0.8847
	TS-m6A-DL	Liver	81.51	85.47	76.56	0.6227	0.8853
	im6APred	Liver	81.44	86.66	76.22	0.63	0.8991
Mouse	iRNA-m6A	Brain	78.26	77.20	79.41	0.57	0.8613
	m6A-TS-CNN	Brain	78.48	86.22	70.74	0.5765	0.8722
	TS-m6A-DL	Brain	78.77	82.91	74.62	0.5774	0.8725
	im6APred	Brain	79.50	83.18	75.83	0.59	0.8832
	iRNA-m6A	Heart	71.30	70.52	72.13	0.43	0.7878
	m6A-TS-CNN	Heart	73.59	75.82	71.36	0.4723	0.8161
	TS-m6A-DL	Heart	75.02	79.31	70.72	0.5023	0.8234
	im6APred	Heart	75.91	82.55	69.27	0.52	0.8377
	iRNA-m6A	Kidney	79.31	78.37	80.32	0.59	0.8697
	m6A-TS-CNN	Kidney	80.76	80.52	81.00	0.6151	0.8855
	TS-m6A-DL	Kidney	80.76	84.21	77.32	0.6168	0.8892
	im6APred	Kidney	81.78	81.96	81.60	0.64	0.9007
	iRNA-m6A	Liver	68.79	67.82	69.86	0.38	0.7620
	m6A-TS-CNN	Liver	71.57	75.56	67.58	0.4328	0.7927
	TS-m6A-DL	Liver	72.03	78.05	66.00	0.4438	0.7913

	im6APred	Liver	78.61	91.34	65.88	0.59	0.8848
	iRNA-m6A	Testis	73.54	72.19	75.08	0.47	0.8182
	m6A-TS-CNN	Testis	76.16	83.45	68.87	0.5288	0.8467
	TS-m6A-DL	Testis	76.44	84.25	68.63	0.5354	0.8432
	im6APred	Testis	77.61	84.49	70.74	0.56	0.8621

Supplementary Note 1

Supplementary Note 1 provides more details regarding actual model configurations used, such as layer sizes, depth, number of parameters, etc. In this study, we selected five different single classifiers among them according to ACC metric as the base classifiers and then produced 21 base-classifier combinations. Besides, we also considered other seven base-classifier combinations generated by same kind of single classifiers. In detail, in according with ACC metric, we selected the best five performing models from the same kind of single classifiers with different hyper-parameters. Finally, a simple average method was employed as ensemble strategy of these 35 base-classifier combinations to classify.

1.SVM
SVC:
Model 1: (C=1.2, probability=True) Optimal parameter
Model 2: (C=0.8, probability=True)
Model 3: (C=1.0, probability=True)
Model 4: (C=1.5, probability=True)
Model 5: (C=2.0, probability=True)

2.KNN
KNeighborsClassifier:
Model 1: (n_neighbors=100) Optimal parameter
Model 2: (n_neighbors=50)
Model 3: (n_neighbors=75)
Model 4: (n_neighbors=150)
Model 5: (n_neighbors=200)

3.RF
RandomForestClassifier:
Model 1: (n_estimators=500) Optimal parameter
Model 2: (n_estimators=250)
Model 3: (n_estimators=400)
Model 4: (n_estimators=650)
Model 5: (n_estimators=600)

4.XGBoost
GradientBoostingClassifier:
Model 1: (n_estimators=250) Optimal parameter
Model 2: (n_estimators=100)
Model 3: (n_estimators=200)
Model 4: (n_estimators=400)
Model 5: (n_estimators=500)

5.CNN

Model 1: "sequential" Optimal parameter

Layer (type)	Output Shape	Param #
conv1d (Conv1D)	(None, 38, 32)	1056
conv1d_1 (Conv1D)	(None, 35, 32)	4128
max_pooling1d (MaxPooling1D)	(None, 17, 32)	0
flatten (Flatten)	(None, 544)	0
dropout (Dropout)	(None, 544)	0
dense (Dense)	(None, 20)	10900
dense_1 (Dense)	(None, 2)	42

Total params: 16,126
Trainable params: 16,126
Non-trainable params: 0

Model 2: "sequential"

Layer (type)	Output Shape	Param #
conv1d (Conv1D)	(None, 38, 32)	1056
conv1d_1 (Conv1D)	(None, 35, 16)	2064
max_pooling1d (MaxPooling1D)	(None, 17, 16)	0
flatten (Flatten)	(None, 272)	0
dropout (Dropout)	(None, 272)	0
dense (Dense)	(None, 20)	5460
dense_1 (Dense)	(None, 2)	42

Total params: 8,622
Trainable params: 8,622
Non-trainable params: 0

Model 3: "sequential"

Layer (type)	Output Shape	Param #
conv1d (Conv1D)	(None, 40, 32)	544

conv1d_1 (Conv1D)	(None, 39, 16)	1040
max_pooling1d (MaxPooling1D)	(None, 19, 16)	0
flatten (Flatten)	(None, 304)	0
dropout (Dropout)	(None, 304)	0
dense (Dense)	(None, 20)	6100
dense_1 (Dense)	(None, 2)	42

Total params: 7,726
Trainable params: 7,726
Non-trainable params: 0

Model 4: "sequential"

Layer (type)	Output Shape	Param #
conv1d (Conv1D)	(None, 40, 32)	544
conv1d_1 (Conv1D)	(None, 39, 32)	2080
max_pooling1d (MaxPooling1D)	(None, 19, 32)	0
flatten (Flatten)	(None, 608)	0
dropout (Dropout)	(None, 608)	0
dense (Dense)	(None, 20)	12180
dense_1 (Dense)	(None, 2)	42

Total params: 14,846
Trainable params: 14,846
Non-trainable params: 0

Model 5: "sequential"

Layer (type)	Output Shape	Param #
conv1d (Conv1D)	(None, 38, 32)	1056
conv1d_1 (Conv1D)	(None, 35, 32)	4128
max_pooling1d (MaxPooling1D)	(None, 17, 32)	0
flatten (Flatten)	(None, 544)	0
dropout (Dropout)	(None, 544)	0

dense (Dense)	(None, 10)	5450
dense_1 (Dense)	(None, 2)	22

===
Total params: 10,656
Trainable params: 10,656
Non-trainable params: 0

6.FCN
Model 1: "sequential"   Optimal parameter

Layer (type)	Output Shape	Param #
flatten (Flatten)	multiple	0
dropout (Dropout)	multiple	0
dense (Dense)	multiple	84224
dense_1 (Dense)	multiple	16448
dense_2 (Dense)	multiple	780
dense_3 (Dense)	multiple	26

===
Total params: 101,478
Trainable params: 101,478
Non-trainable params: 0

Model 2: "sequential"

Layer (type)	Output Shape	Param #
flatten (Flatten)	multiple	0
dropout (Dropout)	multiple	0
dense (Dense)	multiple	84224
dense_1 (Dense)	multiple	32896
dense_2 (Dense)	multiple	8256
dense_3 (Dense)	multiple	780
dense_4 (Dense)	multiple	26

===
Total params: 126,182
Trainable params: 126,182

Non-trainable params: 0

Model 3: "sequential"

Layer (type)	Output Shape	Param #
flatten (Flatten)	multiple	0
dropout (Dropout)	multiple	0
dense (Dense)	multiple	84224
dense_1 (Dense)	multiple	32896
dense_2 (Dense)	multiple	1548
dense_3 (Dense)	multiple	26

Total params: 118,694
Trainable params: 118,694
Non-trainable params: 0

Model 4: "sequential"

Layer (type)	Output Shape	Param #
flatten (Flatten)	multiple	0
dropout (Dropout)	multiple	0
dense (Dense)	multiple	84224
dense_1 (Dense)	multiple	3084
dense_2 (Dense)	multiple	26

Total params: 87,334
Trainable params: 87,334
Non-trainable params: 0

Model 5: "sequential"

Layer (type)	Output Shape	Param #
flatten (Flatten)	multiple	0
dropout (Dropout)	multiple	0
dense (Dense)	multiple	42112
dense_1 (Dense)	multiple	1548

dense_2 (Dense)	multiple	26

Total params: 43,686
Trainable params: 43,686
Non-trainable params: 0

7.LSTM
Model 1: "sequential" Optimal parameter

Layer (type)	Output Shape	Param #
bidirectional (Bidirectional	multiple	10496
bidirectional_1 (Bidirection	multiple	24832
max_pooling1d (MaxPooling1D)	multiple	0
flatten (Flatten)	multiple	0
dropout (Dropout)	multiple	0
dense (Dense)	multiple	25620
dense_1 (Dense)	multiple	42

Total params: 60,990
Trainable params: 60,990
Non-trainable params: 0

Model 2: "sequential"

Layer (type)	Output Shape	Param #
bidirectional (Bidirectional	multiple	3200
bidirectional_1 (Bidirection	multiple	6272
max_pooling1d (MaxPooling1D)	multiple	0
flatten (Flatten)	multiple	0
dropout (Dropout)	multiple	0
dense (Dense)	multiple	12820
dense_1 (Dense)	multiple	42

Total params: 22,334
Trainable params: 22,334

Non-trainable params: 0

Model 3: "sequential"

Layer (type)	Output Shape	Param #
bidirectional (Bidirectional	multiple	3200
bidirectional_1 (Bidirection	multiple	16640
max_pooling1d (MaxPooling1D)	multiple	0
flatten (Flatten)	multiple	0
dropout (Dropout)	multiple	0
dense (Dense)	multiple	25620
dense_1 (Dense)	multiple	42

Total params: 45,502
Trainable params: 45,502
Non-trainable params: 0

Model 4: "sequential"

Layer (type)	Output Shape	Param #
bidirectional (Bidirectional	multiple	10496
bidirectional_1 (Bidirection	multiple	10368
max_pooling1d (MaxPooling1D)	multiple	0
flatten (Flatten)	multiple	0
dropout (Dropout)	multiple	0
dense (Dense)	multiple	12820
dense_1 (Dense)	multiple	42

Total params: 33,726
Trainable params: 33,726
Non-trainable params: 0

Model 5: "sequential"

Layer (type)	Output Shape	Param #
bidirectional (Bidirectional	multiple	37376

bidirectional_1 (Bidirection	multiple	41216
max_pooling1d (MaxPooling1D)	multiple	0
flatten (Flatten)	multiple	0
dropout (Dropout)	multiple	0
dense (Dense)	multiple	25620
dense_1 (Dense)	multiple	42

===

Total params: 104,254
Trainable params: 104,254
Non-trainable params: 0
